# Synthesis and characterization of a new reusable calix[4]arene-bonded silica gel sorbent for antidiabetic drugs†

**DOI:** 10.1039/d2ra04530c

**Published:** 2022-09-05

**Authors:** Fahimeh Hokmabadi, Reza Zadmard, Mohammad Reza Jalali, M. Saeed Abaee

**Affiliations:** Department of Organic Chemistry, Chemistry and Chemical Engineering Research Center of Iran P. O. Box 14335-186 Tehran Iran zadmard@ccerci.ac.ir +98-21-44787785 +98-21-44787719

## Abstract

A novel calix[4]arene-bonded silica gel (C4BS) is prepared by covalent attachment of a calix[4]arene derivative to silica gel through a thiol–ene process. The structure and properties of C4BS were studied by Fourier Transform Infra-Red (FTIR) spectroscopy, thermal gravimetric analysis (TGA), elemental analysis (CHN), scanning electron microscopy (SEM), and surface area analysis (BET). In addition, the binding affinity of some antidiabetic drugs towards C4BS was investigated, by quantitative measurement of the drugs in aqueous solution using UV-visible spectroscopy. Results showed that C4BS has higher affinities than plain silica gel for binding to empagliflozin, dapagliflozin and linagliptin at neutral pH, while metformin hydrochloride was not adsorbed efficiently using either C4BS or plain silica gel. Thus, C4BS can be introduced as a promising binder for selective adsorption of the quoted antidiabetic drugs in pharmaceutical effluents, while being reusable by aqueous/acetonitrile (1 : 1) extraction.

## Abstract 2

C4BS is a new reusable calix[4]arene-bonded silica gel adsorbent for dapagliflozin (DAPA), empagliflozin (EMPA) and linagliptin (LINA) as antidiabetic drugs.

## Graphical abstract

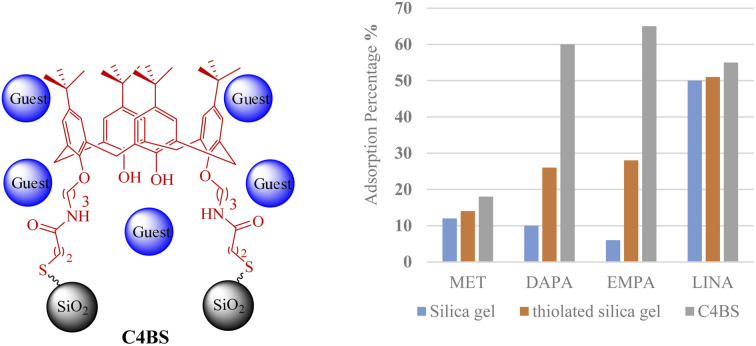

## 1. Introduction

Pharmaceutical and personal care products are present in industrial effluents.^1^ Rapidly growing pharmaceutical and related industrial activities have led to discharging copious amounts of organic, inorganic, biodegradable, and non-biodegradable disposals into the environment.^2^ Recently, pharmaceuticals have been identified as “emerging pollutants” harming seriously water streams and causing significant hazard to aquatic life systems and human beings.^3^ Contamination of the environment with pharmaceutical compounds occurs not only through usage and inappropriate disposals, but also by various production facilities. Different classes of drugs, such as antibiotics, anti-acids, steroids, antidepressants, analgesics, anti-inflammatories, antipyretics, beta-blockers, lipid-lowering drugs, tranquilizers and antidiabetics have been recognized as environmental pollutants.^4,5^

Diabetes mellitus is a metabolic disorder associated with chronic hyperglycemia and occurs as a result of increase in the level of blood sugar.^6^ The disease is classified into insulin-dependent (type-1) and non-insulin dependent (type-2) diabetes. To control hyperglycemia in type-2 diabetes, several therapeutic non-insulin hypoglycemic agents are currently employed.^7^ In this context, metformin hydrochloride (MET) is nowadays considered as a prominent treatment for type-2 diabetes. On the other hand, those diagnosed with high glycated hemoglobin, are usually treated with mixed therapy to achieve glycemic goals.

**Fig. 1:**
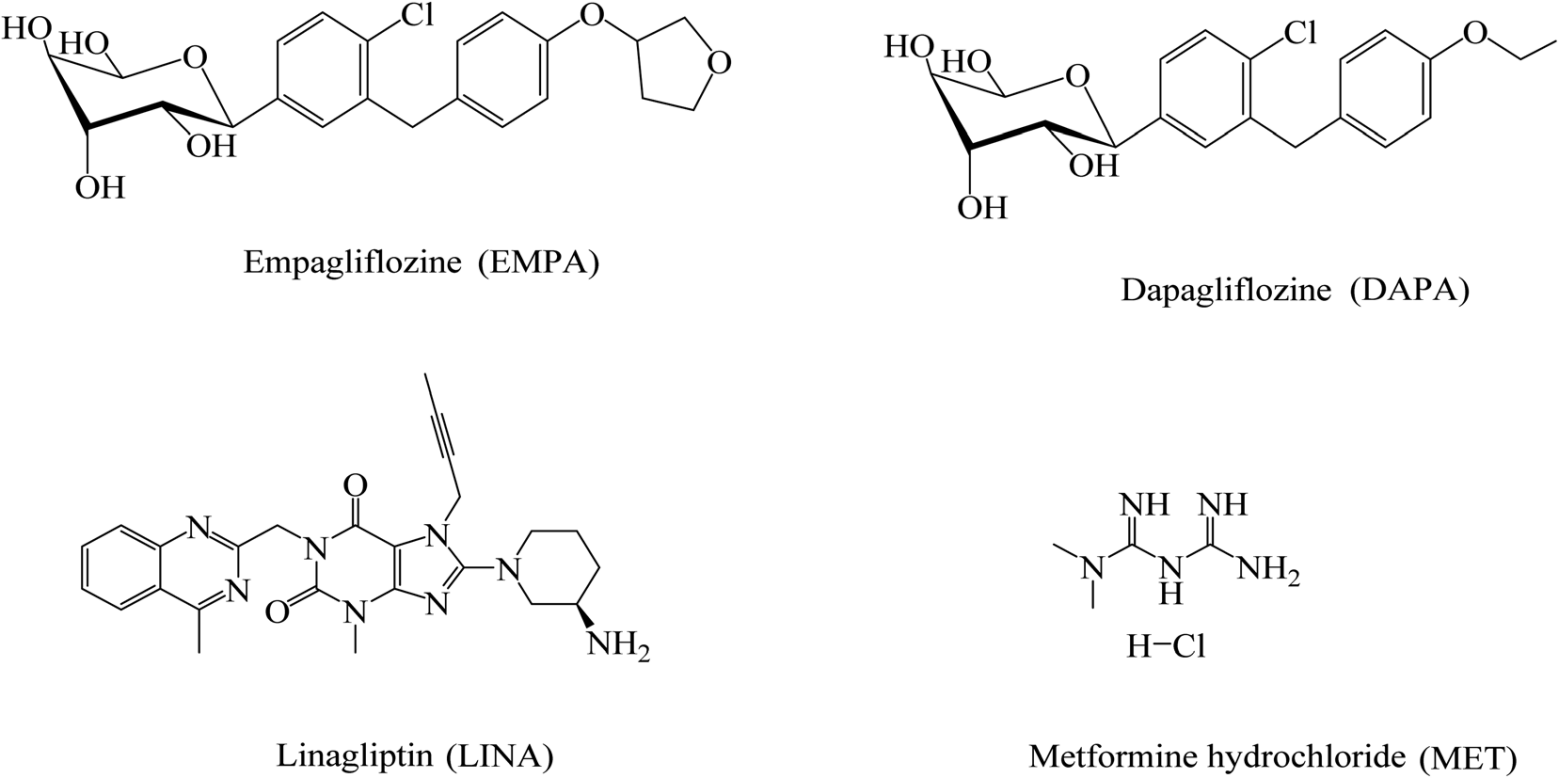
Chemical structures of EMPA, DAPA, LINA and MET.

In recent years, novel therapeutic agents such as dapagliflozin (DAPA), empagliflozin (EMPA) and linagliptin (LINA) are developed for the treatment of type 2 diabetes,^8,9^ and can be used in combination with MET.^10,11^ The chemical structures of these drugs are presented in Fig. 1. A major environmentally related concern is that about 3–5% of the human population are estimated to suffer from diabetes type 2. Thus, the use of considerable amounts of anti-diabetic medications for their treatment would lead to the release of high quantities of anti-diabetic drugs and their metabolites into the environment.^5^

Calixarene is a well-known motif in supramolecular chemistry,^12^ a macrocyclic specie consisted of aromatic units linked by alkylidene moieties.^13^ Due to unique geometry that calixarenes possess, and the availability of active functional groups, calixarenes have found various synthetic and chemical applications.^14,15^ Many investigations are carried out on the adsorption performance of chemically bonded calixarene to silica gel.^16,17^ A notable example is the modification of a silica resin by *p-tert*-butyl calix[4]arene, where the resulting sorbent is conveniently applied for selective binding to azo dyes.^18,19^ In another study, a chlorosulfonyl calix[4]arene attached to silica gel was used as the sorbent of rubber chemical additive.^20^ Moreover, calix[4]arene derivatives covalently attached to mesoporous silica *via* a diisocyanate were utilized to examine the sorption properties of some organotin compounds.^21^ Based on these backgrounds, we were persuaded to design a new calix[4]arene-bonded silica gel, with tendency to selectively bind to DAPA, EMPA and LINA, and thus to be used for separation of such drugs from pharmaceutical effluents.

## 2. Materials and methods

### 2.1 Materials

All chemicals and solvents were purchased from Merck Millipore, Germany and Sigma-Aldrich, USA. Silica gel was purchased from Fluka, USA. All materials were used as received without further purification.

### 2.2 Instrumentation

Melting points were determined with a Buchi B-545 apparatus. ^1^H NMR and ^13^C NMR spectra were recorded on a Bruker-ARX 500 spectrometer. FTIR spectra were recorded on a Bruker Vector 22 infrared spectrometer. The morphology of the polymers was investigated by scanning electron microscopy (TESCAN, Vega3), after the surfaces were coated with gold. The specific surface area, total pore volume and average pore diameter were measured by N_2_ adsorption–desorption method, using a Belsorp mini II instrument at −196 °C. Thermogravimetric analysis (TGA) experiments were carried out using a Netzsch instruments thermal analyzer TGA/209 F 1 Iris. Elemental analyses were performed using CHNS elemental analyzer model: GmbH vario el III. UV-visible absorptions were recorded on a PerkinElmer Lambda 35 spectrophotometer.

### 2.3 Synthesis of 5,11,17,23-tetra-*tert*-butyl-25,27-bis(*N*-acrylo-3-aminopropoxy)-26,28-dihydroxy-calix[4]arene (5)

**Scheme 1:**
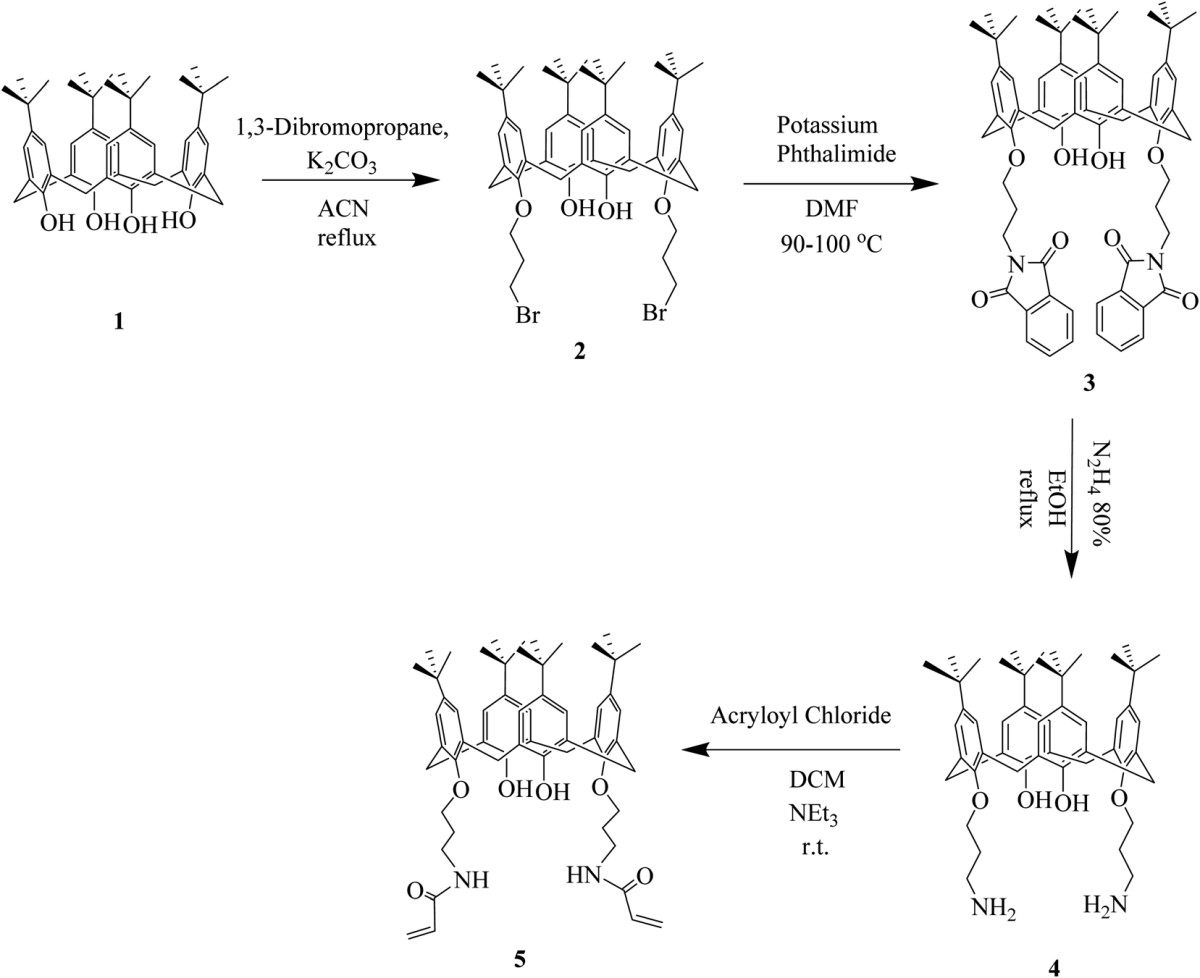
Synthesis of precursor 5.

We have reported the synthesis of calixdiamine 4 previously (Scheme 1).^22^ Acryloyl chloride (0.16 mL, 2.0 mmol) and triethylamine (0.35 mL, 2.0 mmol) were added to a solution of 4 (0.8 g, 1.0 mmol) in anhydrous THF (10 mL). The mixture was stirred under nitrogen atmosphere at −10 °C for 2 h and mixing was continued for another 24 h at room temperature. The mixture was concentrated under reduced pressure at 70 °C, the precipitate was dissolved in CH_2_Cl_2_ and was washed twice with water. The organic layer was dried with sodium sulfate and then was concentrated under reduced pressure to give a solid residue. The solid was recrystallized from methanol to give 0.57 g of 5, as a light-yellow powder: yield 65%; mp: 135–137 °C; ^1^H NMR: (500 MHz, CDCL_3_, TMS), *δ* (ppm), 1.12 (18H, s, C(CH_3_)_3_), 1.31 (18H, s, C(CH_3_)_3_), 2.24 (4H, m, OCCH_2_), 3.39 (4H, d, (CH_2_) eq-bridge, *J* = 12.1), 4.25 (4H, d, (CH_2_) ax-bridge, *J* = 12.1), 3.81 (4H, unresolved t, NCH_2_), 4.15 (4H, unresolved t, OCH_2_), 5.68 (2H, d, CH-olefin, *J* = 9.3), 6.41 (4H, m, CH_2_–olefin), 6.81 (4H, s, CH aromatic), 7.23 (4H, s, CH aromatic), 7.29 (2H, s, NH–amid), 7.61 (2H, s, OH–phenol); ^13^C NMR: (125 MHz, CDCl_3_), *δ* (ppm), 152.08, 149.27, 133.16, 129.65, 129.10, 128.31, 125.18, 76.44, 32.23, 31.22, 28.40, 27.39, 19.40.

### 2.4 Immobilization of calix[4]arene derivative 5 onto the silica gel (C4BS)

**Scheme 2:**
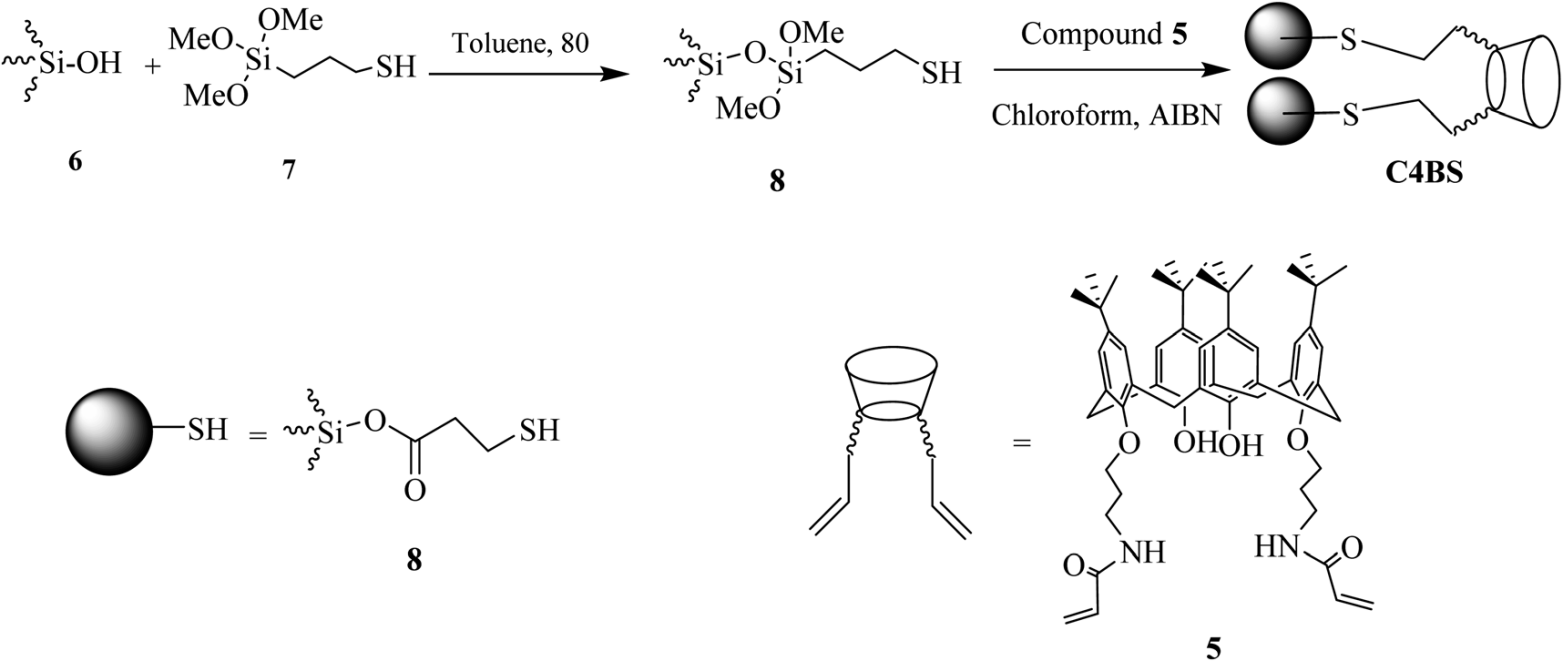
Synthetic pathway of C4BS.

Mesoporous silica gel (1.5 g) was activated by drying in an oven under vacuum at 140 °C for 24 h to remove adsorbed water and maximize the number of surficial silanol groups. The process of immobilization was carried out in a 100 mL round-bottom flask, equipped with a reflux condenser and a gas inlet tube. The activated silica gel (1.5 g) was reacted with 3-(trimethoxysilyl)-1-propanthiol 7 in toluene for 18 h at 80 °C to afford compound 8.^23^ The mixture of 8 and 5 was allowed to reflux in chloroform in the presence of AIBN (azobis-isobutyronitril) for 8 h to obtain C4BS. Since changes in the special surface area of silica gel may result in crash of its particles, no mechanical stirring was used in this reaction. Instead, the stirring was performed by bubbling of a nitrogen stream over the reaction mixture. At the end of the process, the suspension was filtered under vacuum, using a sintered glass funnel and the residue was washed sequentially with CH_2_CL_2_ (20 mL), diethyl ether (20 mL) and methanol (20 mL). The unreacted 3-(trimethoxysilyl)-1-propanthiol was removed with methanol at reflux for 6 h using a Soxhlet system. The final product was dried under vacuum at 110 °C for 6 h. The synthetic pathway to C4BS is summarized in Scheme 2.

### 2.5 Adsorption study

**Fig. 2:**
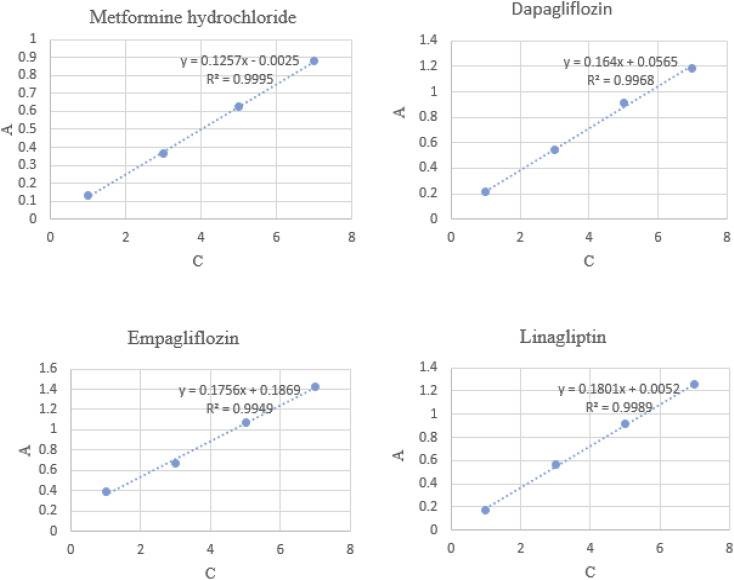
Calibration curves of MET, DAPA, EMPA and LINA.

**Fig. 3:**
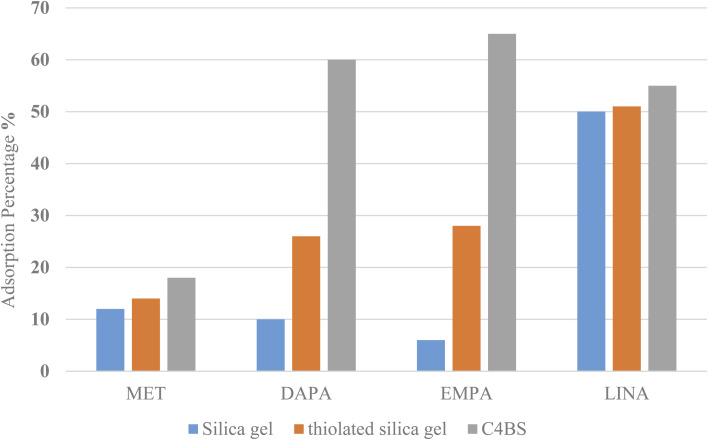
Drugs adsorption percentages onto C4BS in comparison with that of thiolated and pure silica gel.

Adsorption of MET, DAPA, EMPA and LINA was studied *via* batch adsorption experiments on glass columns (1.0 cm i.d. and 15.0 cm length), fitted with a sinter glass at the bottom and shaken at 300 rpm for 10 min. Each column was loaded with 10 mL solutions (10^−5^ M) of either MET, DAPA, LINA or EMPA and known weights of C4BS as the sorbent (100 mg). To illustrate the promoting effect of C4BS, parallel experiments were also carried out using thiolated and pure silica gel. All adsorption experiments were performed in duplicates at room temperature. UV absorptions of the solutions were measured, while being passed through the columns for each sample to calculate the initial and the final concentrations using calibration curves (Fig. 2). The adsorption percentages were calculated using the eqn (1), where *C*_i_ (M) and *C*_f_ (M) are the initial and the final concentrations of each solution before and after adsorption, respectively. The results are shown in Table 1. Fig. 3 compares the adsorption percentage of silica gel, thiolated silica gel and C4BS.

1 % adsorption = (*C*_i_ − *C*_f_)/*C*_i_ × 100

**Table 1:** Adsorption percentages of MET, DAPA, EMPA and LINA onto C4BS in comparison with that of thiolated and pure silica gel

	MET	DAPA	EMPA	LINA
*λ*_max_ (nm)	233	222	223	296
Adsorption% on silica gel	12	10	6	50
Adsorption% on thiolated silica gel	14	26	28	51
Adsorption% on C4BS	18	60	65	55

## 3. Results and discussion

### 3.1 Synthesis and characterization

**Fig. 4:**
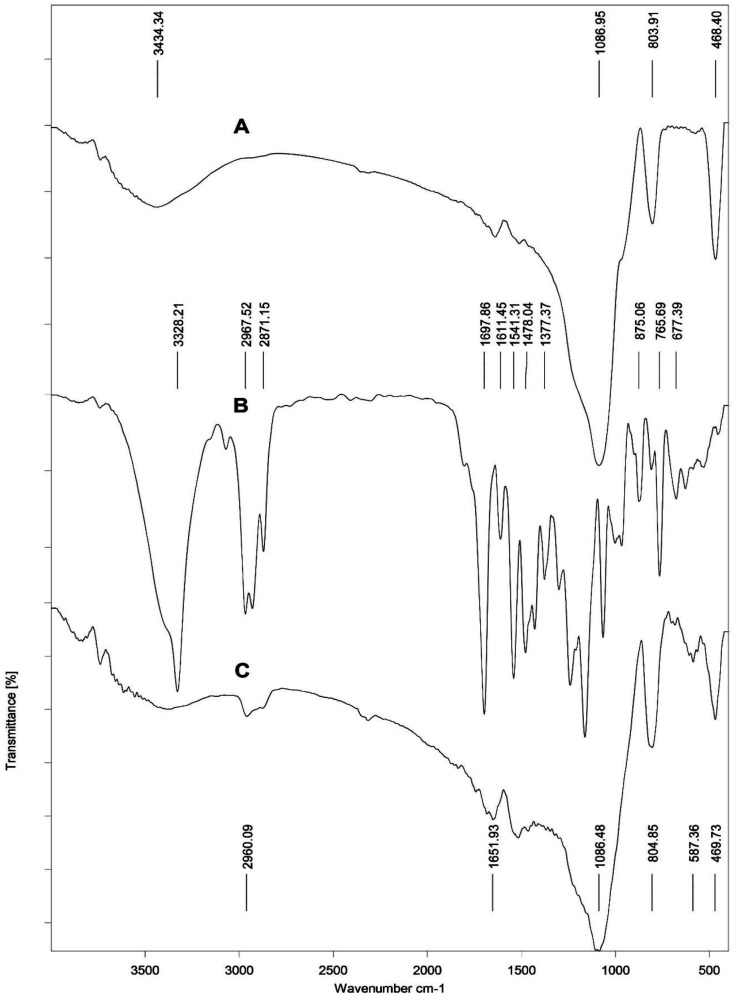
FT-IR spectra of (A) silica gel, (B) compound 5 and (C) C4BS.

In this work, we synthesized a new calix[4]arene-bonded silica gel (C4BS) sorbent and studied the binding properties of the sorbent towards some antidiabetic drugs. For this reason, *p-tert*-butyl calix[4]arene was functionalized at the lower rim to get compound 4. Subsequently, 4 was reacted with acryloyl chloride to prepare 5. Then 5 was loaded onto thiolated silica gel (compound 8) to obtain C4BS. Formation of compound 5 was confirmed by ^1^H NMR and ^13^C NMR spectra (ESI†). Immobilization of 5 onto the surface of silica gel was confirmed by elemental analysis (CHN), FTIR, SEM, surface area analysis (BET) and TGA. Elemental analysis (C, 15.23%; H, 1.5%; N, 0.48%) indicated a loading of 167 μmol of 5 per 1.0 g of silica gel. Fig. 4 shows the FT-IR spectra of the bare silica gel, compound 5 and C4BS. The major peaks related to the bare silica gel are a large broad band about 3430 cm^−1^, indicating the presence of the OH stretching frequency of surficial silanol groups, an intense peak at about 1085 cm^−1^, related to the anti-symmetric Si–O–Si stretching of amorphous silica gel, and a band at about 800 cm^−1^, attributed to the symmetric Si–O–Si stretching. Additionally, FT-IR of 5 showed two characteristic peaks in relation with the aromatic substructure vibrations at about 1478 (C=C aromatic stretching) and about 875 cm^−1^ (C–H aromatic out-of-plane), due to the presence of phenyl groups. Moreover, in the spectrum of C4BS, the appearance of two peaks at about 1651 and 2960 cm^−1^ could be attributed to the absorption bands associated with the amide and the aliphatic C–H groups of the calixarene moiety, respectively. A similar set of bands related to the silica gel was observed as well. The results support that the immobilization has occurred successfully.

**Fig. 5:**
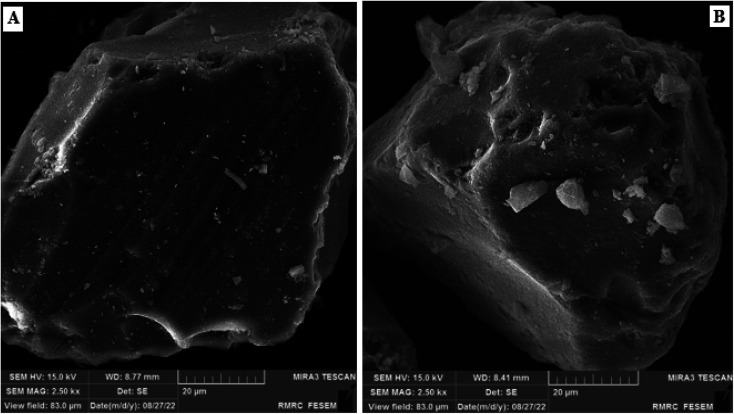
The SEM of (A) silica gel (B) C4BS.

The morphology and the particle size of silica gel after and before immobilization were studied by SEM (Fig. 5) and the average particle diameter was calculated. Results suggest that C4BS was approximately 10 μm larger than bare silica gel, and it appears that immobilization alters the particle size to some extents.^20^

**Fig. 6:**
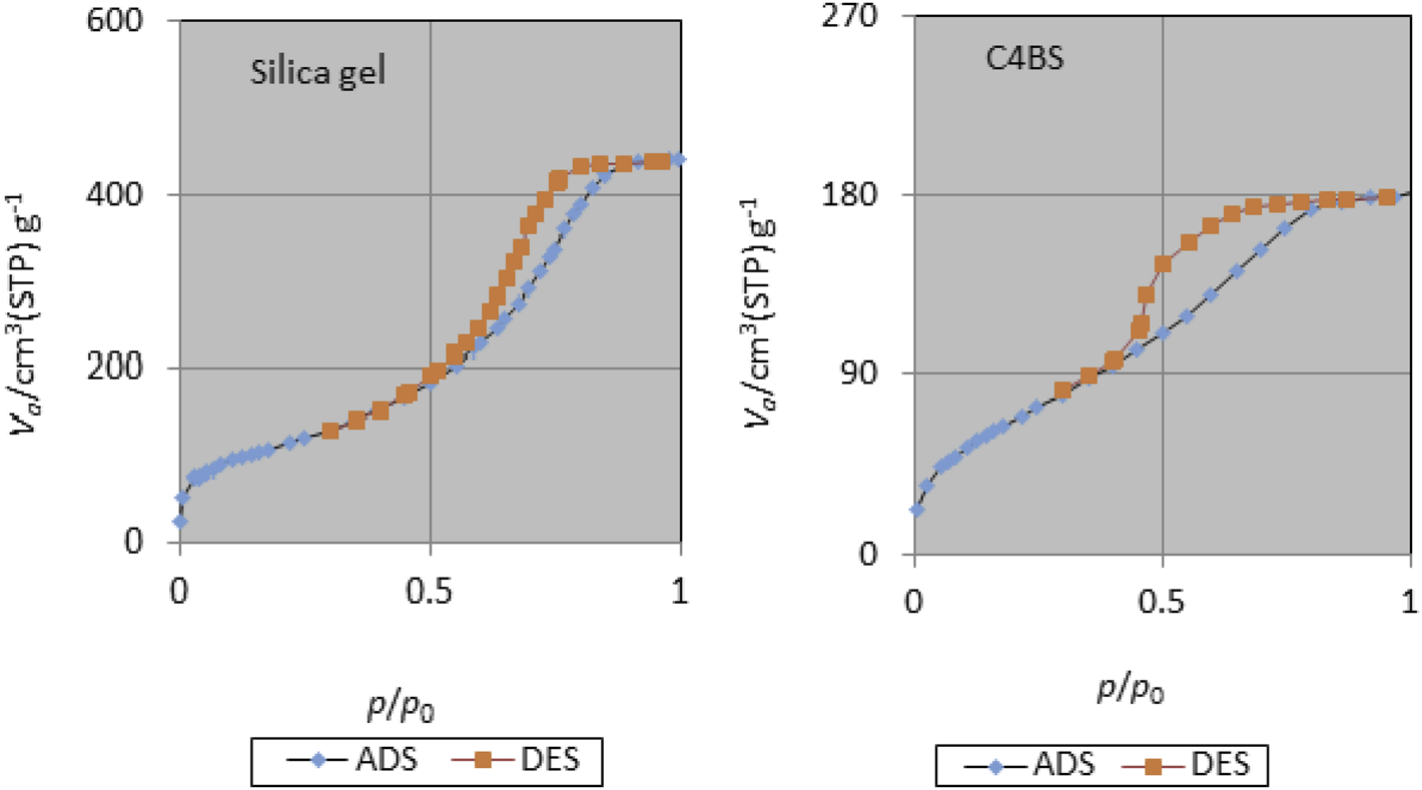
N_2_ adsorption–desorption curves of silica gel and C4BS.

**Fig. 7:**
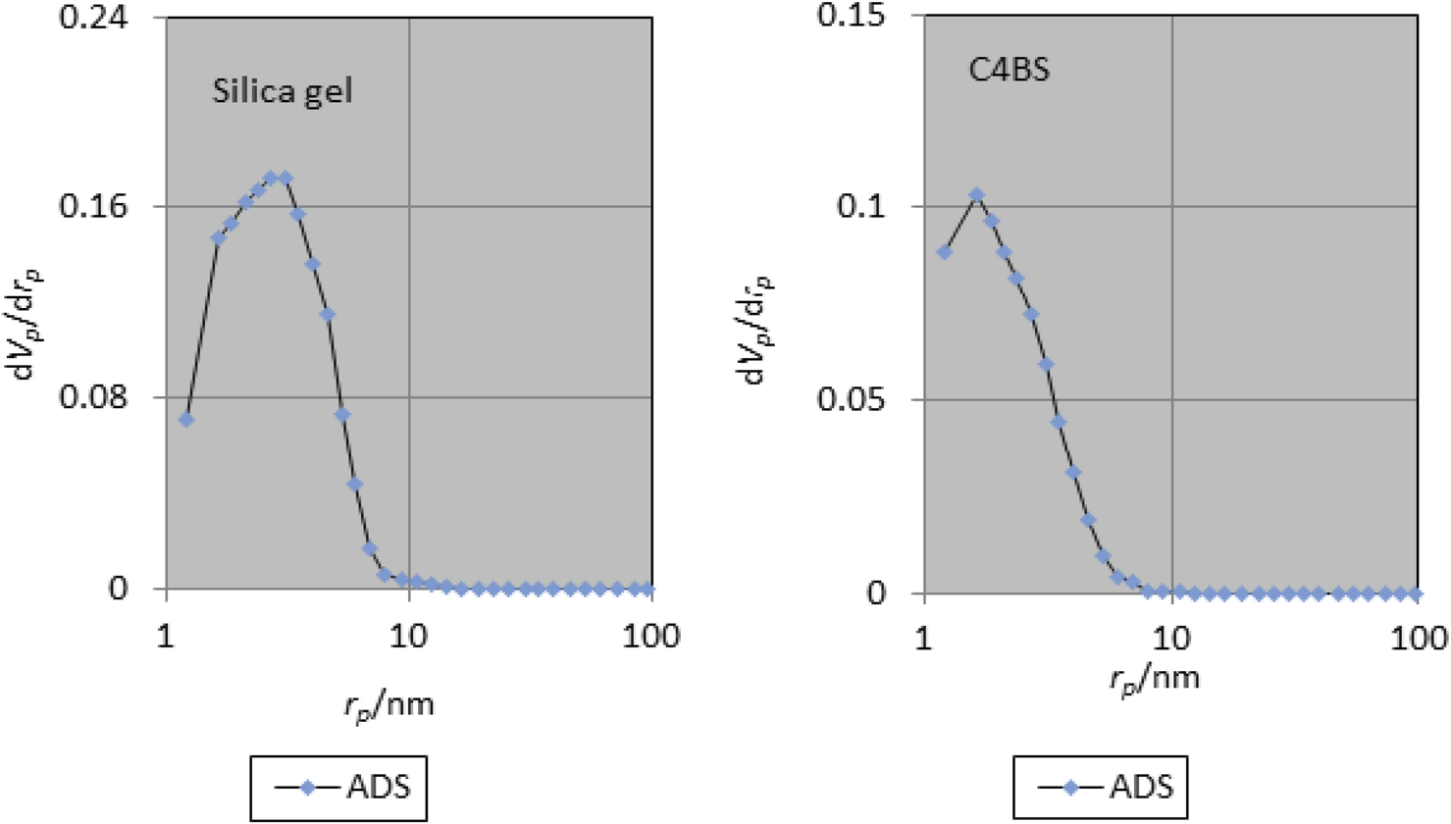
Pore size distribution curves of silica gel and C4BS.

The specific surface area, average pore diameter and total pore volume of bare silica gel and C4BS are 406 m^2^ g^−1^, 6.69 nm, 0.68 cm^3^ g^−1^, and 256 m^2^ g^−1^, 4.35 nm, and 0.28 cm^3^ g^−1^, respectively (Table 2). The decrease in pore diameter, pore volume and surface area of C4BS clearly supports that the immobilization has been occurred.^20^Fig. 6 and 7 show the nitrogen adsorption–desorption and the pore size distribution curves of the bare silica gel and C4BS, respectively. Both cases illustrate type IV adsorption isotherms, showing a hysteresis loop. In other words, the hysteresis in the desorption branch clearly shows the presence of mesoporosity.^20^

**Table 2:** Brunauer–Emmett–Teller (BET) surface area, average pore diameter and pore volume of silica gel and C4BS

Compound	BET surface area (m^2^ g^−1^)	Average pore diameter (nm)	Pore volume (cm^3^ g^−1^)
Silica gel	406.53	6.6995	0.6809
C4BS	256.3	4.35	0.279

**Fig. 8:**
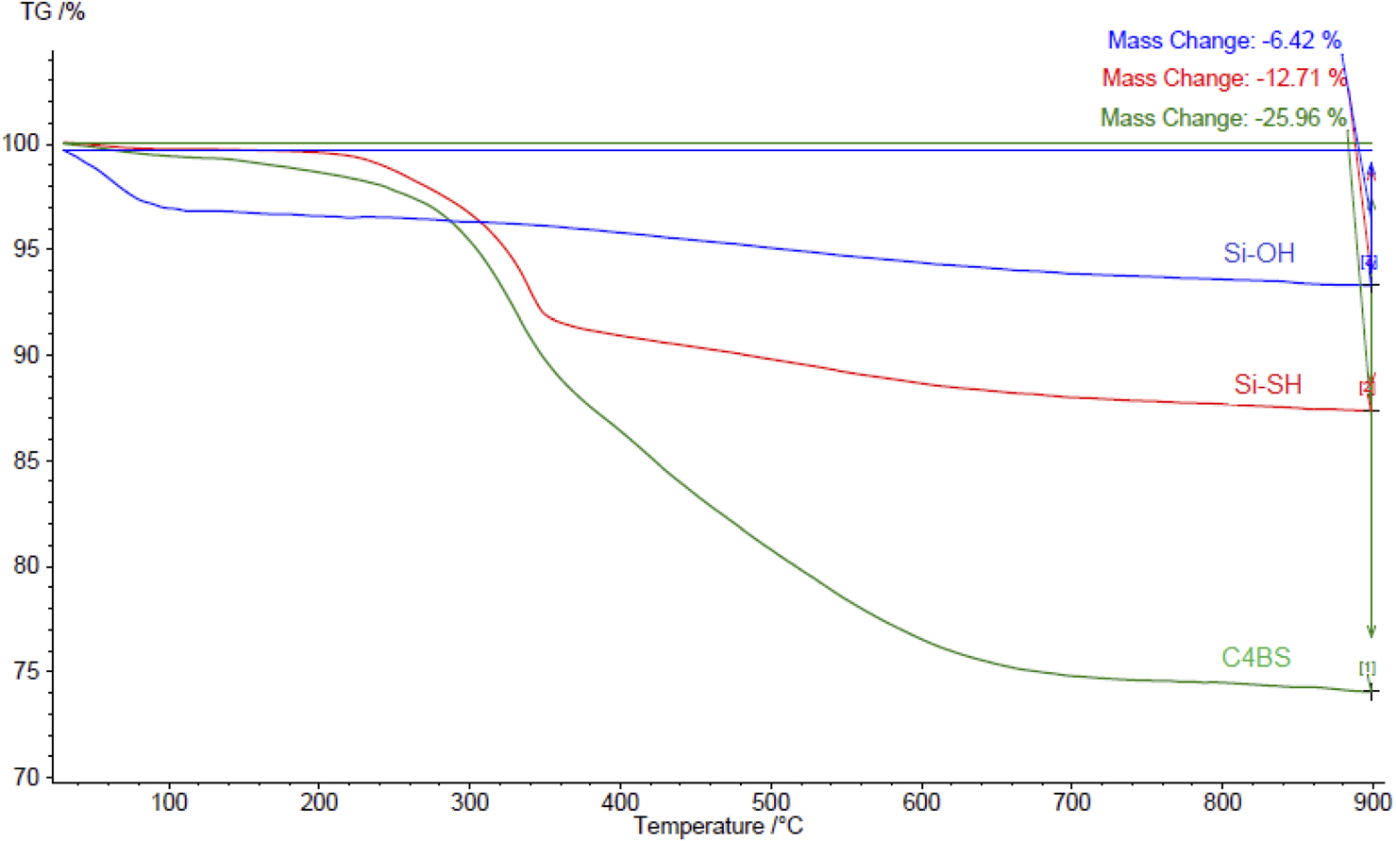
Thermogravimetric curves of bare silica gel, thiolated silica gel and C4BS.

Fig. 8 represents the TGA thermodiagrams of silica gel, thiolated silica gel and C4BS, respectively. The activated silica gel shows a weight loss of 6.42% from 20 to 110 °C, assigned to the physically adsorbed water. Subsequently, a weight loss of 12.7% from 250 to 400 °C assigned to alkyl thiol group is observed. The TGA curve of C4BS shows two stages of weight loss. The first weight loss of 12.7%, between 30 and 350 °C, is attributed to physically adsorbed water and the alkyl thiol group. The second weight loss of approximately 13.2%, observed between 350 and 900 °C, is proportional to decomposition of calixarene, corresponding to 167 μmol of calixarene content per gram of C4BS.

### 3.2 Adsorption behavior study

**Fig. 9:**
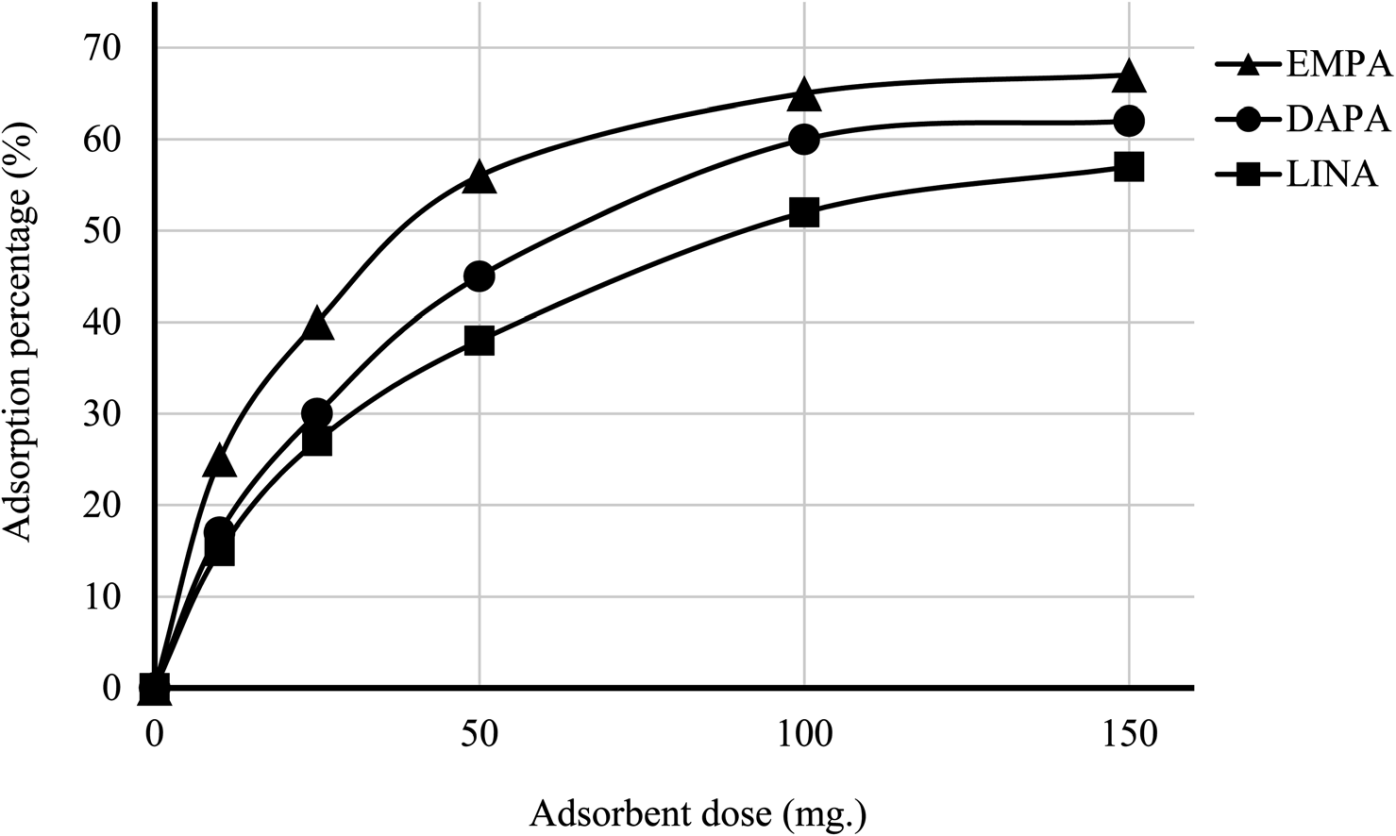
Effect of the amounts of C4BS in the adsorption process.

**Fig. 10:**
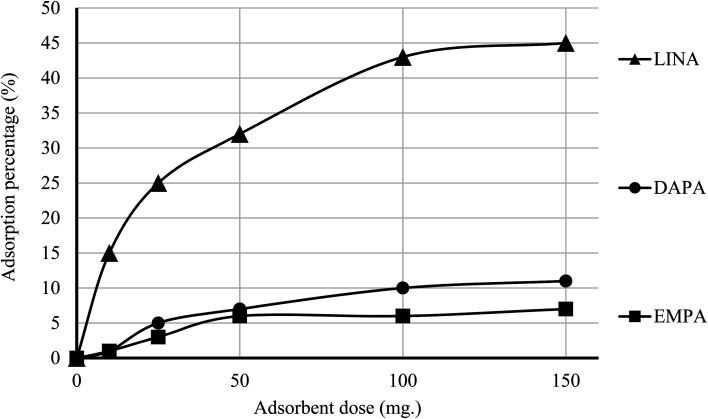
Effect of the amounts of silica gel in the adsorption process.

The efficiency of adsorption is one of the important factors to evaluate the quality of mesoporous silica materials as appropriate adsorbents. For this purpose, batch adsorption experiments were carried out to investigate the adsorption of EMPA, DAPA, LINA and MET onto the materials (C4BS, bare and thiolated silica gel) in aqueous solution with the concentration of (10^−5^ M). It was observed that with an increase in sorbent dosage, sorption was increased, due to the availability of more active surface area to adsorb external particles, attaining the maximum value at 0.1 g of adsorbent. Results show that 0.1 g is sufficient to uptake maximum amounts of EMPA, DAPA and LINA. Using these optimized conditions, adsorption efficiency of bare silica was checked, as represented in Fig. 9 and 10. As it is shown, the adsorption percentages of EMPA, DAPA and LINA increased on C4BS compared to bare silica gel. The better results were obtained at 0.1 g of adsorbents and the adsorption percentages of C4BS were 65%, 60% and 55% for EMPA, DAPA and LINA, respectively. These results suggested that after the modification of silica gel with calix[4]arene derivative 5, the larger adsorption of the cited drugs occurred.

### 3.3 Reusability study

From environmental and economic points of view, reusability is a very important feature for efficiency of the used adsorbents to lower the load of pollutants in sewage and disposal systems. No need to mention that it is also necessary to minimize or omit the release of drugs into the environment. In this study, the adsorbents were first loaded with drugs and the desorption process was then carried out under the optimum conditions. For this reason, after each cycle, the adsorbent was extracted with a 1 : 1 water: acetonitrile mixture at 50 °C for 2 h to remove the surface-bound drugs. The recovery experiments showed that approximately 90% of the drugs are recovered.

## 4. Conclusion

The present study demonstrates that some of the anti-diabetic drugs can be effectively adsorbed using C4BS. Results of adsorption experiment show that C4BS is more efficient than bare silica gel. It has been observed that 0.1 g of C4BS is able to remove 65%, 60% and 55% of EMPA, DAPA and LINA from the solutions, respectively. It is possible that the calixarene aromatic cavity attached to silica gel provides several electrons rich sites on silica gel, establishing more interactions with drug molecules compared to bare silica gel. Additionally, C4BS was reusable and can be easily recovered. Therefore, C4BS promising to outperform bar silica gel in terms of elimination of EMPA, DAPA and LINA from pharmaceutical disposal streams.

## Conflicts of interest

There are no conflicts to declare.

## Supplementary Material

RA-012-D2RA04530C-s001
