# Supplementary material for: Synthesis and characterization of a new reusable calix[4]arene-bonded silica gel sorbent for antidiabetic drugs
Source: RSC Adv. 2022 Sep 5;12(39):25123–32. doi: 10.1039/d2ra04530c (PMC9443680; doi:10.1039/d2ra04530c)
Supplement: RA-012-D2RA04530C-s001 [file RA-012-D2RA04530C-s001.pdf]

Electronic Supplementary Material (ESI) for RSC Advances.
This journal is © The Royal Society of Chemistry 2022

Synthesis and characterization of a new reusable calix[4]arene-bonded silica gel sorbent for antidiabetic drugs

Fahimeh Hokmabadi, Reza Zadmard, * M. Reza Jalali and M. Saeed Abaee

Department of Organic Chemistry, Chemistry and Chemical Engineering Research Center of Iran, P.O. Box 14335-186, Tehran, Iran

Correspondence e-mail: zadmard@ccerci.ac.ir, Tel: +9821-44787719; Fax: +9821-44787785

SUPPLEMENTARY INFORMATION

5,11,17,23-Tetra-tert-butyl-25,27-bis(N-acrylo-3-aminopropoxy)-26,28-dihydroxy-calix[4]arene (5)

Acryloyl chloride (0.16 mL, 2.0 mmol) and triethylamine (0.35 ml, 2.0 mmol) were added to a solution of **4** (0.8 g, 1.0 mmol) in anhydrous THF (10 ml). The mixture was stirred under nitrogen atmosphere at -10 °C for 2 h and mixing was continued for another 24 h at room temperature. The mixture was concentrated under reduced pressure at 70 °C, the precipitate was dissolved in CH_2Cl_2 and was washed twice with water. The organic layer was dried with sodium sulfate and then was concentrated under reduced pressure to give a solid residue. The solid was recrystallized from methanol to give 0.57 g of **5,** as a light-yellow powder: yield 65%; mp: 251-253 °C; FT-IR (KBr, *ν,* cm^{-1}): 3328, 2968, 2871, 1698, 1611, 1541, 1478, 1377, 1241, 1163, 1066, 968, 875, 766, 677; ^1H NMR: (500 MHz, $CDCL_3$, TMS), δ (ppm), 1.12 (18H, s, $C(CH_3)_3$), 1.31 (18H, s, $C(CH_3)_3$), 2.24 (4H, m, $OCCH_2$), 3.39 (4H, d, (CH_2) eq-bridge, J = 12.1), 4.25 (4H, d, (CH_2) ax-bridge, J = 12.1), 3.81 (4H, unresolved t , NCH_2), 4.15 (4H, unresolved t , OCH_2), 5.68 (2H, d, CH-olefin, J = 9.3), 6.41 (4H, m, CH_2-olefin), 6.81 (4H, s, CH aromatic), 7.23 (4H, s, CH aromatic), 7.29 (2H, s, NH-amid), 7.61 (2H, s, OH-phenol); ^{13}C NMR: (125 MHz, $CDCl_3$), δ (ppm), 152.08, 149.27, 133.16, 129.65, 129.10, 128.31, 125.18, 76.44, 32.23, 31.22, 28.40, 27.39, 19.40.

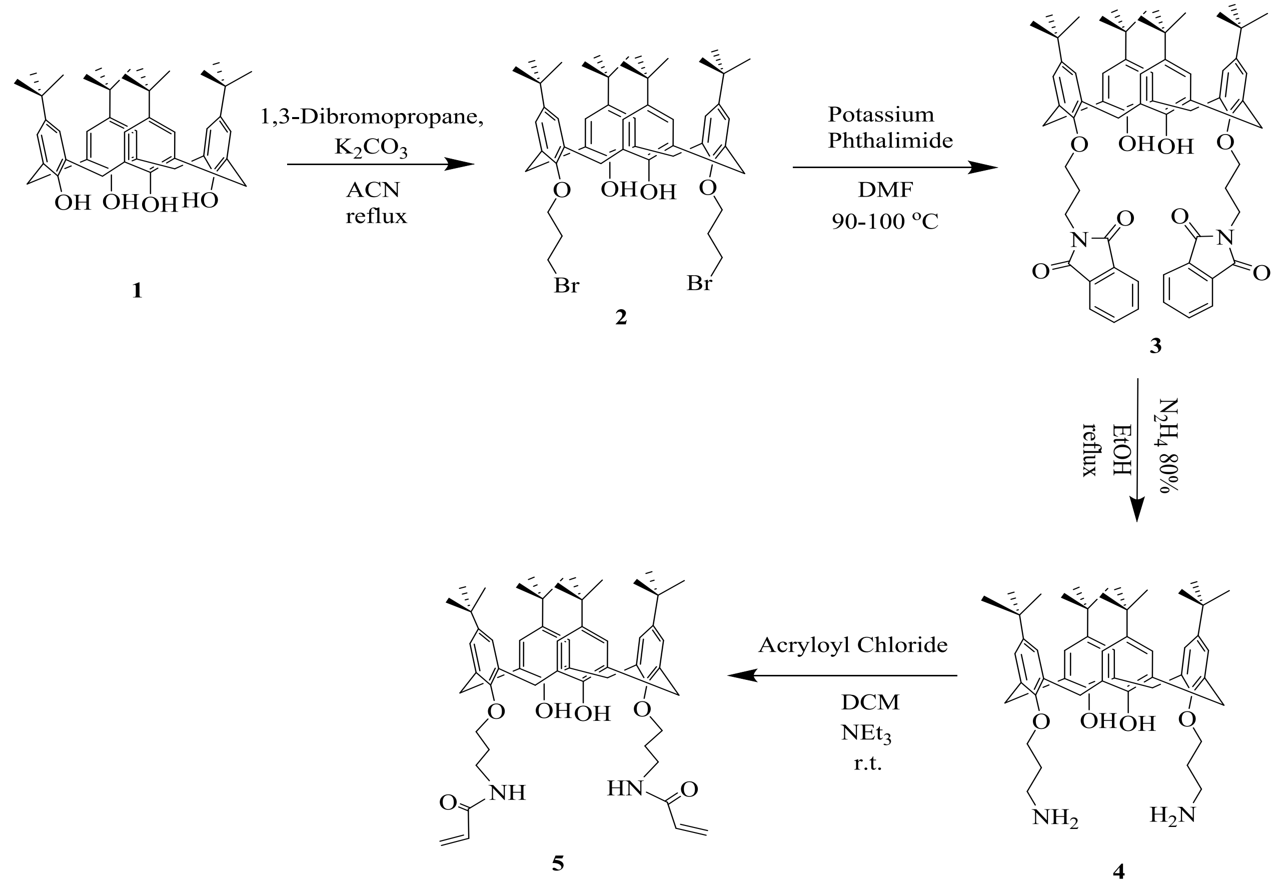
1,3-Dibromopropane,
K2CO3
ACN
reflux
Potassium
Phthalimide
DMF
90-100 °C
N2H4 80%
EtOH
reflux
Acryloyl Chloride
DCM
NEt3
r.t.
OH OH OH HO
OH OH
Br
Br
O
N
NH2
H2N
NH
HN
1
2
3
4
5

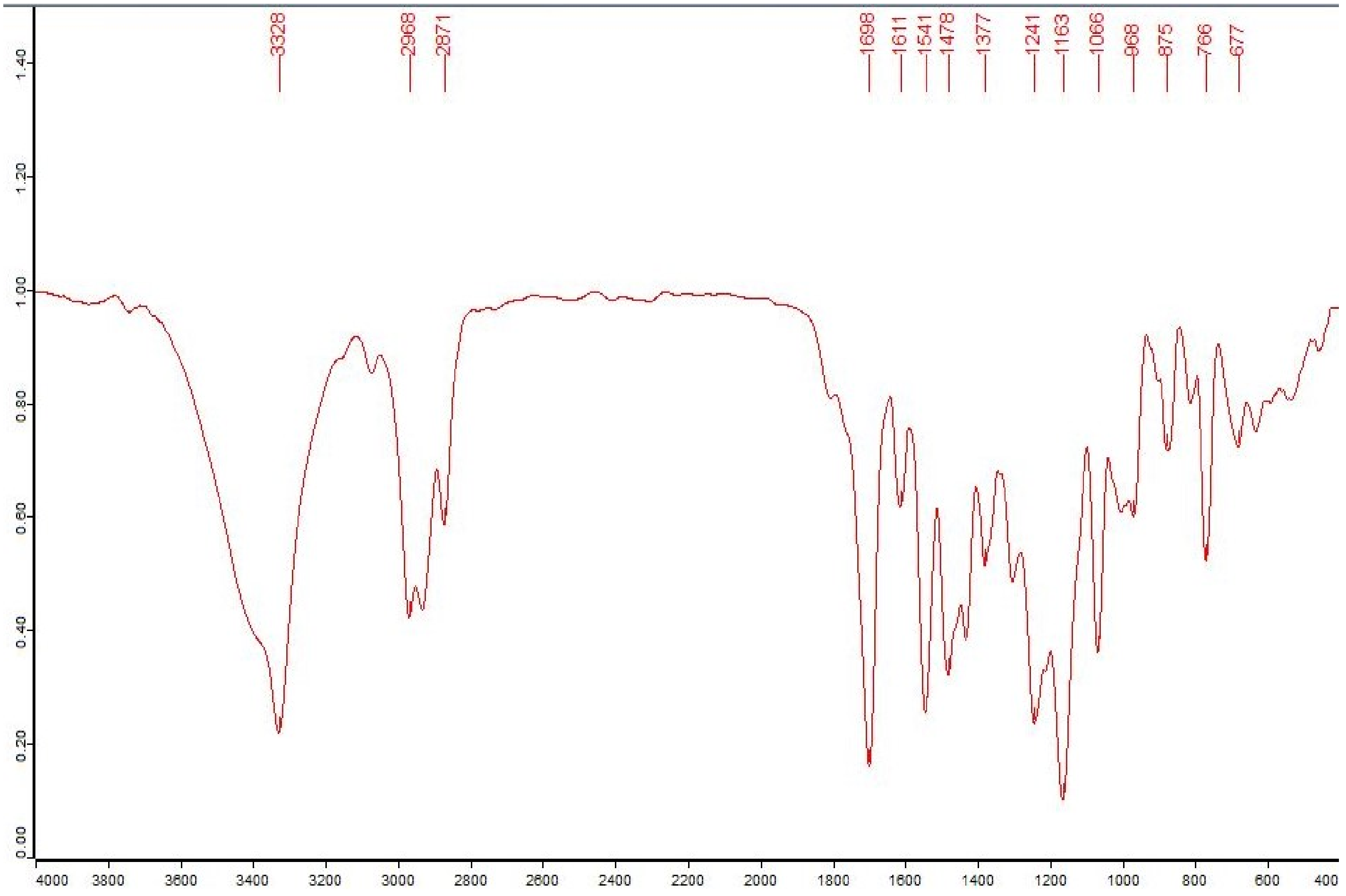
3328
2968
2871
1698
1611
1541
1478
1377
1241
1163
1066
968
875
766
677
1.40
1.20
1.00
0.80
0.60
0.40
0.20
0.00
4000
3800
3600
3400
3200
3000
2800
2600
2400
2200
2000
1800
1600
1400
1200
1000
800
600
400

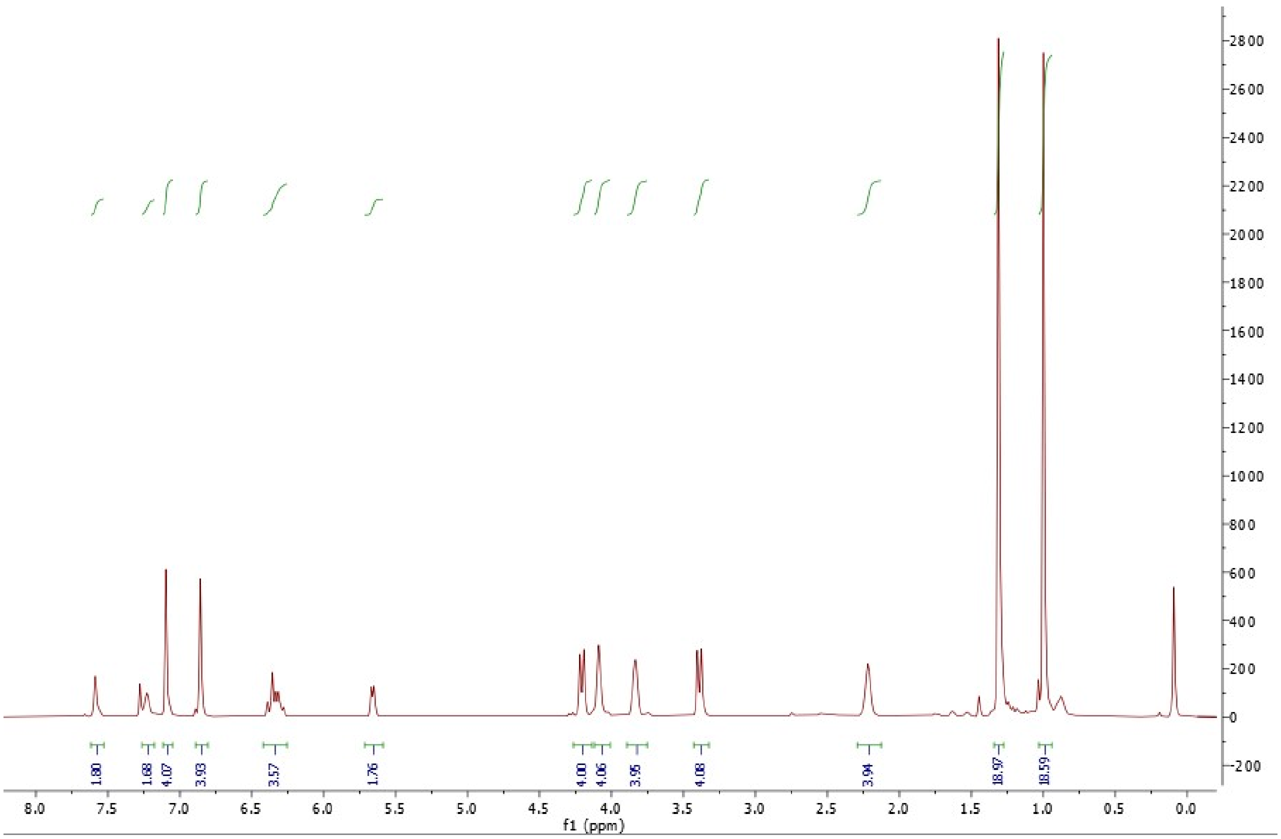

2800
2600
2400
2200
2000
1800
1600
1400
1200
1000
800
600
400
200
0
-200
1.80
1.68
4.07
3.93
3.57
1.76
4.00
4.06
3.95
4.08
3.94
18.97
18.59
8.0
7.5
7.0
6.5
6.0
5.5
5.0
4.5
4.0
3.5
3.0
2.5
2.0
1.5
1.0
0.5
0.0
f1 (ppm)

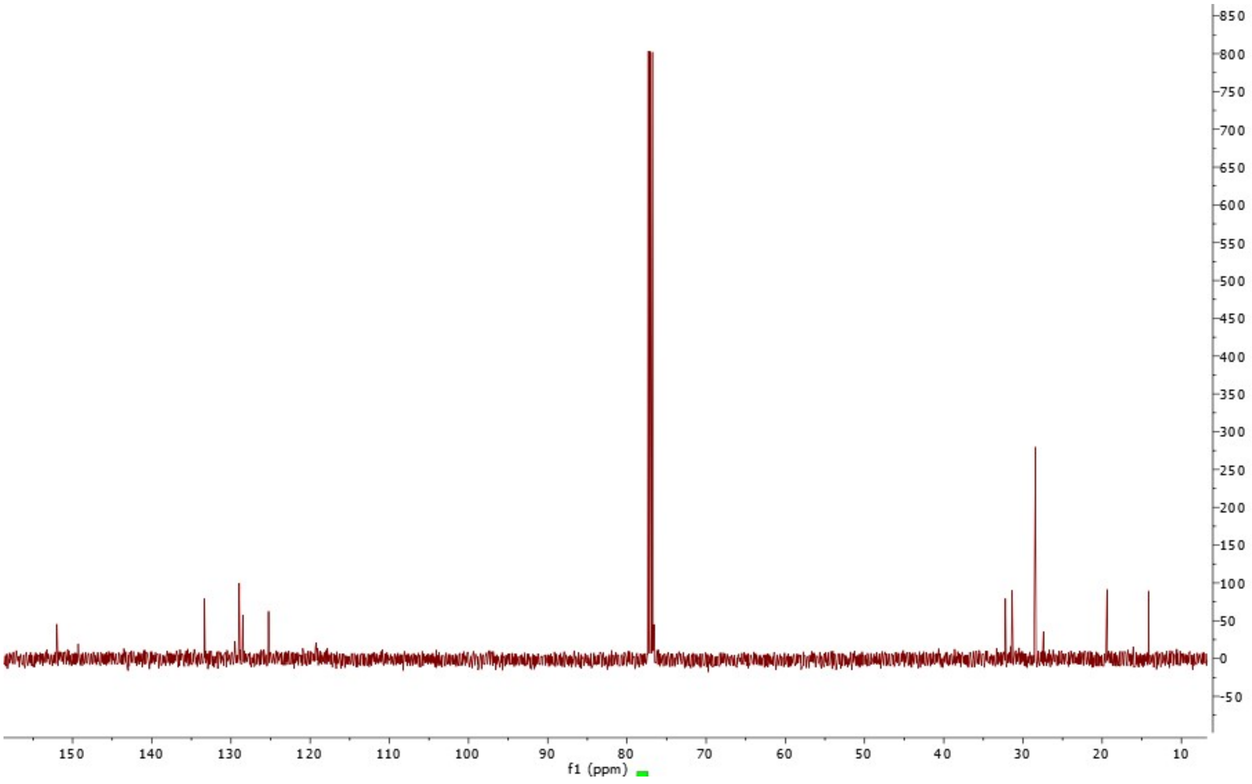

850
800
750
700
650
600
550
500
450
400
350
300
250
200
150
100
50
0
-50
150
140
130
120
110
100
90
80
70
60
50
40
30
20
10
f1 (ppm)
